# Glial cell line-derived neurotrophic factor inhibits mast-cell-like RBL-2H3 cells activation via Ca2+-mediated degranulation and Ca2+/CaMKⅡ/JNK pathway

**DOI:** 10.3389/fphar.2025.1697815

**Published:** 2025-11-18

**Authors:** Wei Huang, Li Zeng, Li Zhang, Xinxing Zhang, Qin Xie

**Affiliations:** Department of Geriatric Medicine and Gastroenterology, Sichuan Provincial People’s Hospital, School of Medicine, University of Electronic Science and Technology of China, Chengdu, China

**Keywords:** glial cell line-derived neurotrophic factor (GDNF), mast cell (MC), Ca2+, inflammatory bowel disease, C-jun N-terminal kinase (JNK)

## Abstract

**Introduction:**

Mast cells are important component of the intestinal immune system, play a crucial role in the pathogenesis of inflammatory bowel disease. Glial cell line-derived neurotrophic factor (GDNF), as a multifunctional growth factor, has recently garnered attention for its role in the inhibition of mast cells activation. This study aims to explore the potential mechanisms by which GDNF inhibits mast cell activation.

**Methods:**

In this study, RBL-2H3 cells were used as an *in vitro* cell model of mast cells, which were cultured and treated with various interventions prior to collection of cells and culture supernatants. IgE-mediated degranulation were evaluated through β-hexosaminidase release assays. Culture supernatants were analyzed for TNFα, IL-1β, and IL-6 secretion using ELISA. Key signaling molecules—GDNF family receptor α1 (GFRα1), receptor Tyrosine Kinase (RET), calcium/calmodulin-dependent protein kinase II (CaMKⅡ), total and phosphorylated c-Jun N-terminal kinase (JNK), and JNK isoforms—were quantified at mRNA and protein levels using Quantitative Real-time polymerase chain reaction and Western blot. Intracellular Ca^2+^ were monitored fluorometrically. Immunofluorescence and protein binding assays were used to confirm interactions between GDNF-GFRα1/RET complexes and CaMKⅡ-JNK associations.

**Results:**

GDNF inhibited the degranulation and release of inflammatory cytokines in activated RBL-2H3 cells. The intracellular Ca^2+^ and the phosphorylation of JNK were reduced in activated RBL-2H3 cells after GDNF treatment. Immunofluorescence results demonstrated co-localization of GDNF with GFRα1 on RBL-2H3 cell membranes and CaMKII with JNK in the cytoplasm. There were interactions between GDNF and GFRα1/RET, as well as CaMK II and JNK. RET inhibitor eliminated this inhibitory effect of GDNF on RBL-2H3 cell degranulation and inflammatory factor release. Ca2+ chelator and CaMKⅡ RNAi had the same inhibitory effect on degranulation, release of inflammatory cytokines and phosphorylation of JNK. However, in their presence, GDNF had no additional inhibitory effect.

**Conclusion:**

GDNF can decrease the intracellular concentration of Ca^2+^ in activated RBL-2H3 cells by attaching to GFLα1/RET receptors located on the membrane of RBL-2H3 cells, subsequently inhibiting Ca^2+^-mediated degranulation and the Ca^2+^/CaMKII/JNK pathway responsible for the release of inflammatory cytokines.

## 1 Introduction

Glial cell line-derived neurotrophic factor (GDNF), as an important neurotransmitter secreted by enteric glial cells (EGCs), plays a role in promoting the survival, proliferation, migration, differentiation, and axonal growth of intestinal neurons (Young et al., 2004). With the deepening research of the role of GDNF in intestinal inflammation, it has gradually evolved from an executor of EGCs to protect the intestinal epithelial barrier (Meir et al., 2019; Meir et al., 2016; Zhang et al., 2010) to play an important role in multiple protective mechanisms against intestinal inflammation (Kearon et al., 2021; Xiaoling et al., 2024; Zeng et al., 2021). It is known that the dysfunction of the intestinal mucosal barrier may only be the first step in the pathogenesis of inflammatory bowel disease (IBD), and the abnormality of intestinal immune function may play a more critical role in the occurrence and development of IBD. Abundance studies have confirmed that abnormalities in the intestinal immune response are closely related to abnormalities in the function of the enteric nervous system (Brinkman et al., 2019; Campaniello et al., 2017). Therefore, further exploring the mechanism of GDNF on intestinal immune function is an important step in clarifying the pathogenesis of IBD.

Mast cells, which are essential components of the immune system, arise from immature bone marrow precursors whose differentiation in peripheral organs is driven by microenvironmental cues (Ribatti and d'Amati, 2023). As secretory immune cells in the gut, intestinal mast cells are considered to be a bridge between the enteric nervous system and the immune system (Wood, 2004). An increasing number of studies have shown that mast cells are crucial for maintaining the barrier function of the gastrointestinal tract. Their interactions with neurons, immune cells, and epithelial cells are associated with various gastrointestinal diseases, especially irritable bowel syndrome (IBS) and IBD (Molfetta et al., 2025). Previous studies have shown that the number of mast cells in the intestines of patients with IBD increase significantly (Vivinus-Nébot et al., 2014; Kurashima et al., 2012). Activated mast cells can trigger and exacerbate intestinal inflammation by releasing various inflammatory mediators such as histamine, tryptase, and cytokines such as tumor necrosis factor-α(TNF-α) and interleukin-6(IL-6) (Beunk et al., 2013; Hamilton et al., 2011; Rijnierse et al., 2006). These studies suggest that activation of mast cells play a very important role in the occurrence and development of IBD. Our previous research found that GDNF was able to reduce the degranulation of intestinal mast cells in SD rats with experimental colitis induced by dextran sulfate sodium salt (DSS), and improve their disease activity index and intestinal tissue damage score (Xie et al., 2020). This effect might have been achieved through lessening intracellular Ca^2+^ levels and downregulating JNK signaling pathway, as both of them are thought to be crucial for mast cell activation. It has been well-established that mast cell degranulation are dependent on the rise in intracellular Ca^2+^ (Ma and Beaven, 2011; Chen et al., 2017). Moreover, It was found that mast cells failed to degranulate efficiently and release IL-1β after stimulation in JNK-deficient mice (Guma et al., 2010). Kim et al. also demonstrated the role of the JNK pathway in mast cell activation, which was supported by their finding that hispidulin inhibits JNK activation and thereby reduces cytokine expression in activated mast cells (Kim et al., 2019). However, the potential mechanism by which GDNF influences the degranulation of intestinal mast cells and downstream pathways to modulate their release of inflammatory cytokines remains unclear. Calcium/calmodulin-dependent protein kinaseⅡ(CaMKⅡ) might be involved in this regulation process of GDNF on intestinal mast cell. On the one hand, the activity of CaMKⅡ is regulated by the intracellular concentration of Ca^2+^ (Rusciano et al., 2010). On the other hand, CaMKⅡ has been proven to promote the phosphorylation of JNK (Han et al., 2020; Tan et al., 2020). The study of Qu et al. may support this hypothesis, as it found that CaMKⅡ could promote asthma through the activation of mast cells (Qu et al., 2017).

Therefore, this study aims to investigate the potential mechanisms by which GDNF regulates mast cell activity, with a particular focus on the effects of GDNF on intracellular Ca2+ and the Ca^2+^/CaMKⅡ/JNK signaling pathway.

## 2 Materials and methods

### 2.1 Cell culture and treatment

RBL-2H3 cells (Pricella Biotechnology Co., Ltd., Wuhan, China) were used as an *in vitro* cell model of mast cells (Falcone et al., 2018), which were cultured in Dulbecco’s Modified Eagle’s Medium (GIBCO, CA, United States) supplemented with 10% fetal bovine serum (GIBCO, CA, United States), 100 U/mL penicillin, and 100 μg/mL streptomycin. Cells were maintained in a humidified incubator at 37 °C with 5% CO2. RBL-2H3 cells were seeded in 96-well plates at a density of 2.5 × 10^4^ cells per well and sensitized by overnight incubation with 1 μg/mL DNP-IgE (Thermo Fisher Scientific, St. Louis, United States). Following sensitization, the cells were subjected to the various interventions detailed below (Table1).

**TABLE 1:** The intervention measures of cells.

Groups	DNP-IgE	GDNF	RET inhibitor*	Ca2+ chelator#	DNP-HSA	CaMKⅡ RNAi
a	1ug/mL	0	0	0	0	0
b	1ug/mL	0	0	0	0.5ug/mL	0
c	1ug/mL	100 ng/mL	0	0	0.5ug/mL	0
d	1ug/mL	100 ng/mL	1uM	0	0.5ug/mL	0
e	1ug/mL	0	0	10uM	0.5ug/mL	0
f	1ug/mL	100 ng/mL	0	10uM	0.5ug/mL	0
g	1ug/mL	0	0	0	0.5ug/mL	1 × 10^7^Tu/mL
h	1ug/mL	100 ng/mL	0	0	0.5ug/mL	1 × 10^7^Tu/mL

Tu, Transduction Units per ml; uM, umol/L. *RET, inhibitor (GSK3179106, MCE); #Ca2+ chelator: BAPTA-AM(MCE, MedChemExpress).

### 2.2 Construction of ShCaMKⅡ plasmid

To construct shRNA plasmids targeting CaMKII, three specific RNAi target sequences (sh1: TCC​TCT​GAG​AGC​ACC​AAC​AT; sh2: GGT​GCC​TAC​GAT​TTC​CCA​TCA; sh3: GCA​GCT​GAT​CGA​AGC​CAT​AAG) were designed and synthesized by Ribobio in China. These sequences were inserted into the PLKO.1-puro vector via AgeI and EcoRI restriction sites. The shRNA oligonucleotides were phosphorylated, annealed, and ligated into the pre-digested vector. Positive clones were identified by polymerase chain reaction (PCR) screening and confirmed by Sanger sequencing. Verified plasmids were purified and prepared for subsequent lentiviral packaging and functional assays.

### 2.3 Measurement of degranulation and inflammatory cytokine release of RBL-2H3 cells

The cells’ grouping was the same as Table 1. After incubation with DNP-HSA for 30 min at 37 °C, the supernatant was collected for the detection of β-hexosaminidase based on a method previously published (Wang et al., 2012). In brief, 50 μL of the supernatant were added to a 96-well plate and mix with 50 μL of substrate solution (1 mM p-nitrophenyl-N-acetyl-β-D-glucosaminide in 0.1 M citrate buffer, pH 5.0). Incubate the plate at 37 °C for 1 h. Stop the reaction by adding 200 μL of 0.1 M carbonate buffer (Na_2_CO_3_/NaHCO_3_, pH 10.0). Measure the absorbance at 405 nm with a microplate reader. The released β-hexosaminidase, serving as a marker of mast cell degranulation, was calculated using the ratio of the absorbance in the supernatant to the combined absorbance of the supernatant and the detergent-solubilized cell pellet. After incubation with DNP-HSA for 60 min at 37 °C, the cytokine levels (TNFα, IL-1β, IL-6) in the supernatant were detected using ELISA kits (R&D Systems, MN, United States) according to the manufacturer’s instructions.

### 2.4 Measurement of intracellular Ca^2+^ concentration

Groups were treated as per Table1. Fluo-4 a.m. (MCE, Shanghai, China), as a fluorescent probe for detecting intracellular Ca^2+^ was added to the cell groups. The cells were incubated at 37 °C for 60 min to load the probe. The culture medium was discarded, and the cells were washed twice with PBS. Finally, after 3 min of incubation with DNP-HSA, fluorescence intensity was immediately measured using a fluorometer (Ex/Em = 488/526 nm).

### 2.5 The expression of relevant signaling molecules in RBL-2H3 cells

Different groups of cells treated as Table1. After incubation with DNP-HSA (at 37 °C for 30 min), RNA and protein were extracted separately. The relevant gene and protein expression of cell signaling pathway in RBL-2H3 cells were detected by qRT-PCR and Western blot (WB): GDNF family receptor α1 (GFRα1), receptor Tyrosine Kinase (RET), CaMKⅡ, total Jun amino-terminal kinase (JNK), phosphorylated Jun amino-terminal kinase (p-JNK), and JNK isoforms (JNK1, 2, 3). All of the antibodies were purchased from abcam (Cambridge, UK). The sequences of the primers are listed in Supplementary Table S1.

### 2.6 Immunofluorescence co-localization assay

To verify the co-localization of GDNF with GFRα1/RET and CaMKⅡ with JNK in RBL-2H3 cells, the immunofluorescence was conducted as follows. RBL-2H3 cells grown on coverslips were fixed with 4% paraformaldehyde for 15–30 min at room temperature, permeabilized with 0.1% Triton X-100 on ice for 5–10 min, and blocked with 5% BSA for 30 min. The cells were then incubated overnight at 4 °C with primary antibodies against GFRα1, GDNF, CaMKII, and JNK (1:500 dilution). After washing, the samples were incubated with Cy3-or FITC-conjugated secondary antibodies for 2 h at 37 °C in the dark. Nuclei were counterstained with DAPI for 30 s at room temperature. Finally, the coverslips were mounted and observed under fluorescence microscope.

### 2.7 Protein binding experiments

#### 2.7.1 Direct protein binding experiment

Using recombinant technology, probe proteins were fused with Glutathione S transferase (GST), and the fusion proteins bound to Glutathione (GSH) immobilized on a carrier through GST. Proteins interacting with the fusion proteins were adsorbed and separated when mixed with the solid-phase complex. GDNF-GST, CaMK II-GST, GFRα1, and JNK expression vectors were constructed, transfected into cells for overexpression, and subsequently cell lysates containing GDNF-GST, CaMK II-GST, GFRα1, and JNK proteins were obtained. GDNF-GST and CaMKⅡ-GST were purified using GSH affinity beads and incubated overnight at 4 °C with GFRα1 and JNK protein lysates. After centrifugation at 1,000 *g* for 3 min at 4 °C, the supernatants were discarded, and the beads were washed five times with lysis buffer. The protein samples were boiled in loading buffer, and the binding of GDNF to GFRα1 and CaMKⅡ to JNK was detected by SDS-PAGE and Western blot analysis.

#### 2.7.2 Indirect protein binding experiment

Co-Immunoprecipitation (Co-IP) was used to study protein interactions based on the specific interaction between antibodies and antigens. This assay allows for the determination of whether two target proteins bind *in vivo*, thereby confirming the physiological interactions of GDNF and GFRα1/RET, as well as CaMKⅡ and JNK in intact cells. Cell lysates were prepared and incubated overnight at 4 °C with magnetic beads bound to antibodies against GDNF or CaMKⅡ. The beads were collected by centrifugation at 1000 g for 3 min at 4 °C, and the supernatants were discarded. The beads were washed five times with lysis buffer by centrifugation, and the protein samples were boiled in loading buffer. The binding of GDNF to GFRα1/RET and CaMKⅡ to JNK was detected by SDS-PAGE and Western blot analysis.

### 2.8 Statistical analysis

All data were presented as the mean ± standard deviation (SD) and analyzed using IBM SPSS 20.0 statistical software. A one-way analysis of variance (ANOVA) was employed for comparing data among groups when multiple groups of data met the assumptions of normality and homogeneity of variance, followed by *post hoc* analysis conducted using Tukey’s HSD test. Otherwise, the Kruskal - Wallis test and Dunn’s *post hoc* test with Bonferroni correction was used. *P < 0.05 was considered statistically significant.

## 3 Results

### 3.1 The effect of GDNF on degranulation and inflammatory cytokines release of RBL-2H3 cells

**FIGURE 1:**
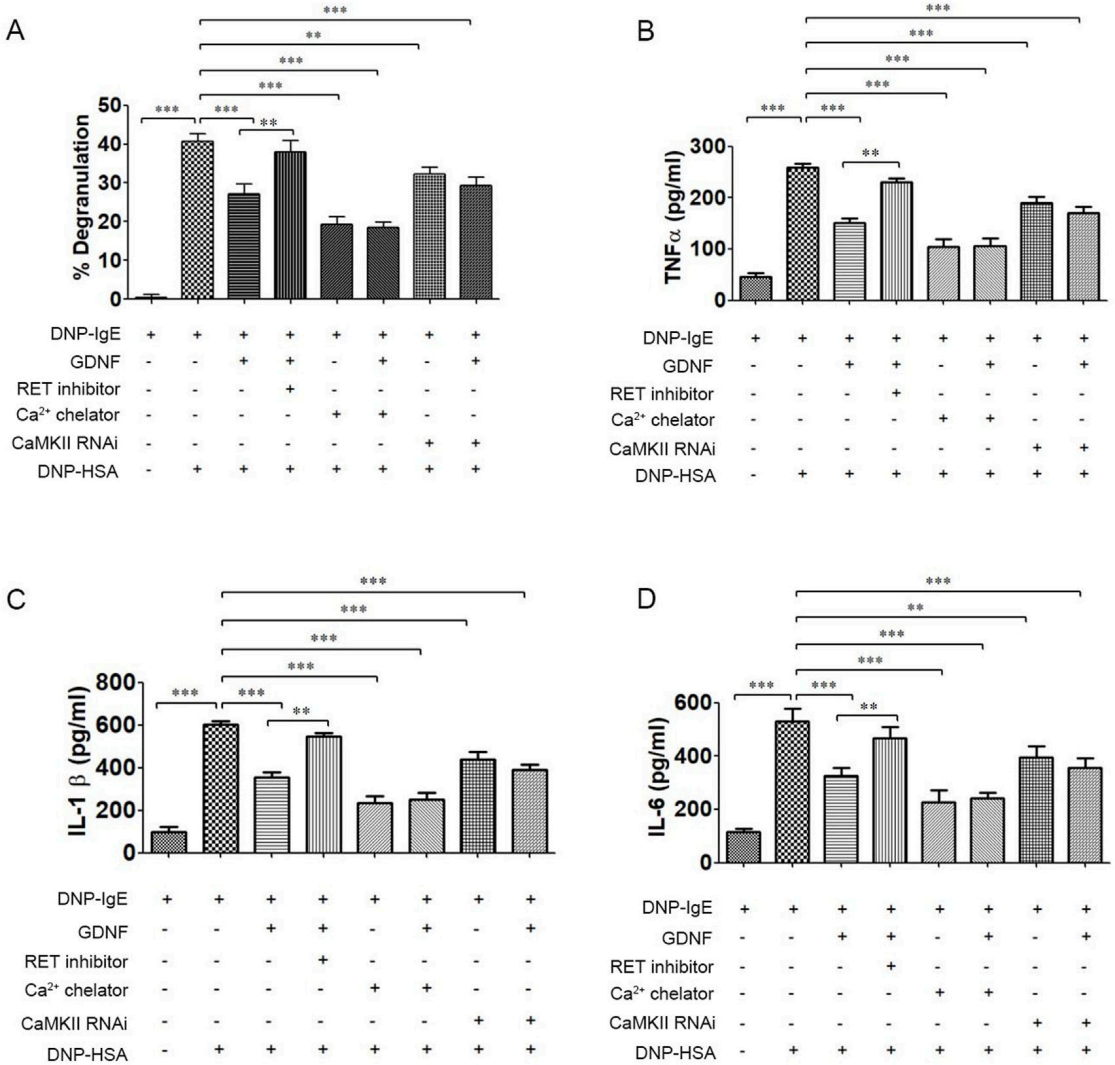
The effect of GDNF on degranulation and inflammatory cytokine release of RBL-2H3 cells. **(A)** The degranulation of RBL-2H3 cells; **(B)** The release of TNFα in RBL-2H3 cells. **(C)** The release of IL-1β in RBL-2H3 cells. **(D)** The release of IL-6 in RBL-2H3 cells. GDNF(100 ng/mL, 30min pre-treatment) significantly reduced β-hexosaminidase and inflammatory cytokines release in RBL-2H3 cells activated by DNP-HSA. This inhibitory effect is abolished by co-treatment with RET inhibitors(1uM); Ca2+ chelator and CaMKII RNAi independently inhibit degranulation and cytokine release in activated RBL-2H3 cells. In presence of Ca2+ chelator and CaMKII RNAi, GDNF had no additional inhibitory effect. ** p < 0.01; *** p < 0.001.

The results showed that GDNF inhibited the degranulation and release of inflammatory cytokines in activated RBL-2H3 cells. RET inhibitors could eliminate the inhibitory effect of GDNF on the activation RBL-2H3 cells. Ca^2+^ chelator and CaMKⅡ RNAi could also inhibit degranulation and the release of inflammatory cytokines from RBL-2H3 cells activation. In their presence, GDNF had no additional inhibitory effect (Figure 1). These results suggest that GDNF exerts its inhibitory effect through a mechanism dependent on RET and upstream of intracellular Ca^2+^ and CaMKII.

### 3.2 The effect of GDNF on the intracellular Ca2+ concentration in RBL-2H3 cells

**FIGURE 2:**
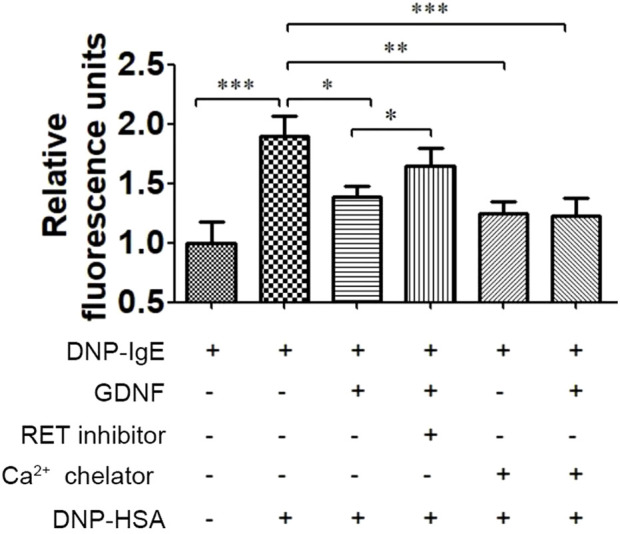
Effect of GDNF on intracellular Ca2+ concentration in RBL-2H3 cells. GDNF (100 ng/mL, 30min pre-treatment) significantly decreased the intracellular Ca^2+^ concentration induced by DNP-HSA (0.5ug/mL, 3 min). This effect was eliminated by RET inhibition. There was no additional reduction was observed when GDNF was co-applied with the Ca2+ chelator. *p < 0.05; **p < 0.01; ***p < 0.001.

As shown in Figure 2, GDNF decreased the intracellular Ca2+ concentration in activated RBL-2H3 cells, and the RET inhibitor could eliminate this inhibitory effect of GDNF. Compared with Ca2+ chelator alone, the combination of Ca2+ chelator and GDNF did not further reduce intracellular Ca2+ levels.

### 3.3 The effect of GDNF on Ca^2+^/CaMKII/JNK pathway in RBL-2H3 cells

**FIGURE 3:**
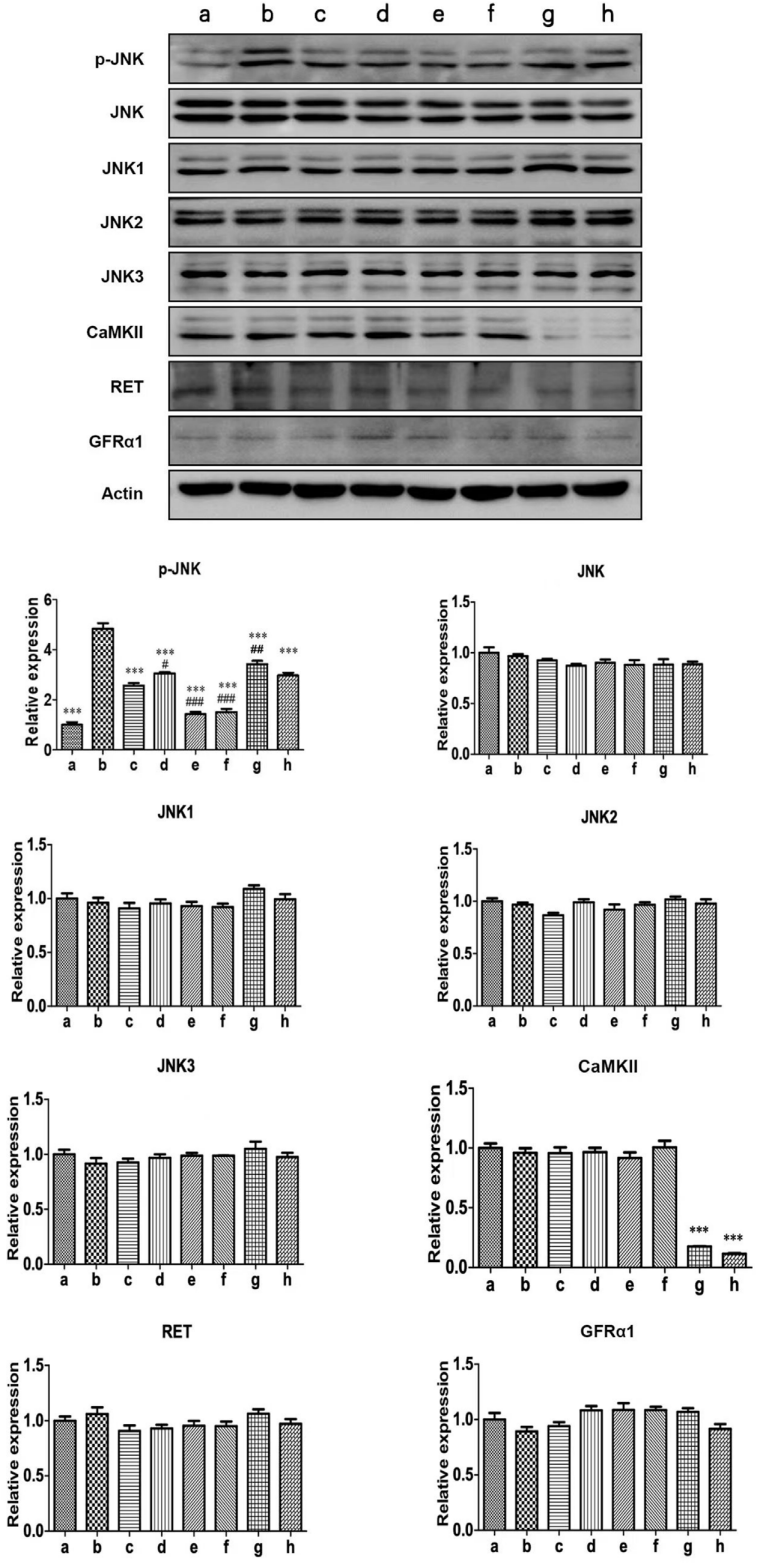
WB detection of related proteins of Ca2+/CaMKⅡ/JNK pathway in RBL-2H3 cells. The results showed that the expression of p-JNK protein in activated RBL-2H3 cells significantly increased (b vs. a), which were suppressed by GDNF (c vs. b), CaMKII RNAi (g vs. b), and the Ca^2+^ chelator (e vs. b). RET inhibitior could alleviate the inhibitory effect of GDNF on the expression of p-JNK protein in activated RHL-2H3 cells (d vs. c), while the Ca^2+^ chelator could further enhance this effect of GDNF (f vs. c). In presence of Ca2+ chelator (e vs. f) and CaMKⅡ RNAi (g vs. h), GDNF had no additional inhibitory effect on the expression of p-JNK protein. Protein levels of total JNK, JNK isoforms (JNK1, 2, 3), GFRα1, RET, and CaMKII showed no significant changes across groups.***p < 0.001 vs. b; #p < 0.05 vs. c; #p < 0.01 vs. C; ###p < 0.001 vs. c.

**FIGURE 4:**
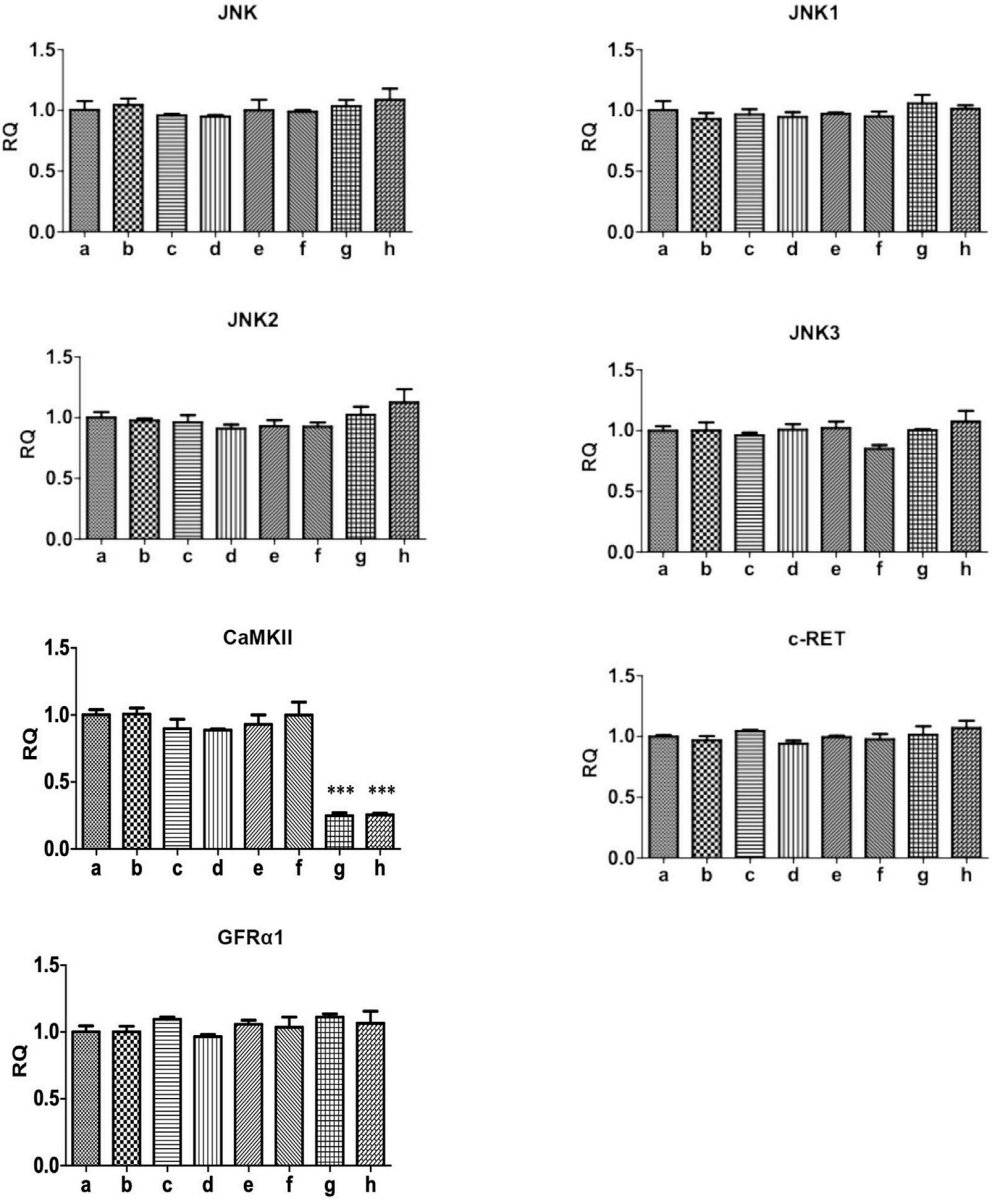
qRT-PCR detection of related genes of Ca^2+^/CaMKⅡ/JNK pathway in RBL-2H3 cells. The results showed that the mRNA expression of total JNK, JNK isoforms (JNK1, 2, 3), GFRα1, RET, and CaMKII showed no statistical significance between the groups.

The protein expression of p-JNK was significantly increased in activated RBL-2H3 cells. GDNF, Ca^2+^ chelator and CaMKII RNAi could decrease the elevated level of p-JNK. RET inhibitior could alleviate the inhibitory effect of GDNF on the expression of p-JNK protein in activated RHL-2H3 cells, while the Ca^2+^ chelator could further enhance this effect of GDNF. In presence of Ca2+ chelator and CaMKⅡ RNAi, GDNF had no additional inhibitory effect on the expression of p-JNK protein. The mRNA and protein expression of total JNK, JNK isoforms (JNK1, 2, 3), GFRα1, RET, and CaMKⅡ showed no statistical significance between the groups (Figures 3, 4).

### 3.4 The co-localization of GDNF with GFRα1 and CaMKII with JNK in RBL-2H3 cells

**FIGURE 5:**
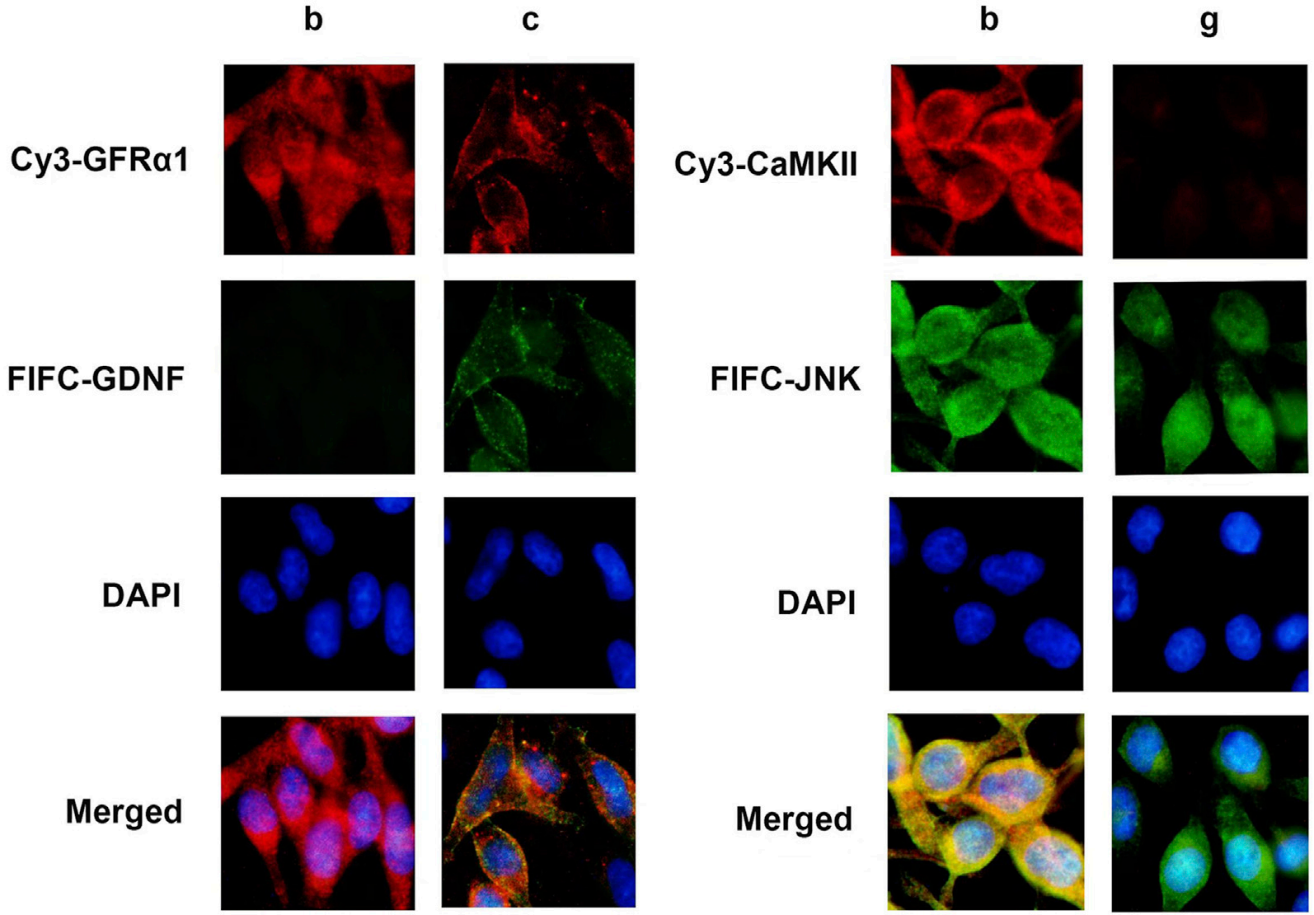
Immunofluorescence staining in RBL-2H3 cells. Immunofluorescence image of condition b and c reveals the subcellular localization of GDNF (green) and GFRα1 (red), with merge indicating co-localization on the cell membrane (yellow). Immunofluorescence image of condition b and c shows CaMKII (green) and JNK (red), with merge indicating co-localization in the cytoplasm (yellow). Nuclei were counterstained with DAPI (blue).

Immunofluorescence results showed that GDNF and GFRα1 were co-localized on the membrane of RBL-2H3 cells, and CaMKII and JNK were co-localized in the cytoplasm (Figure 5).

### 3.5 The interaction between GDNF and GFRα1/RET, CaMKII and JNK in RBL-2H3 cells

**FIGURE 6:**
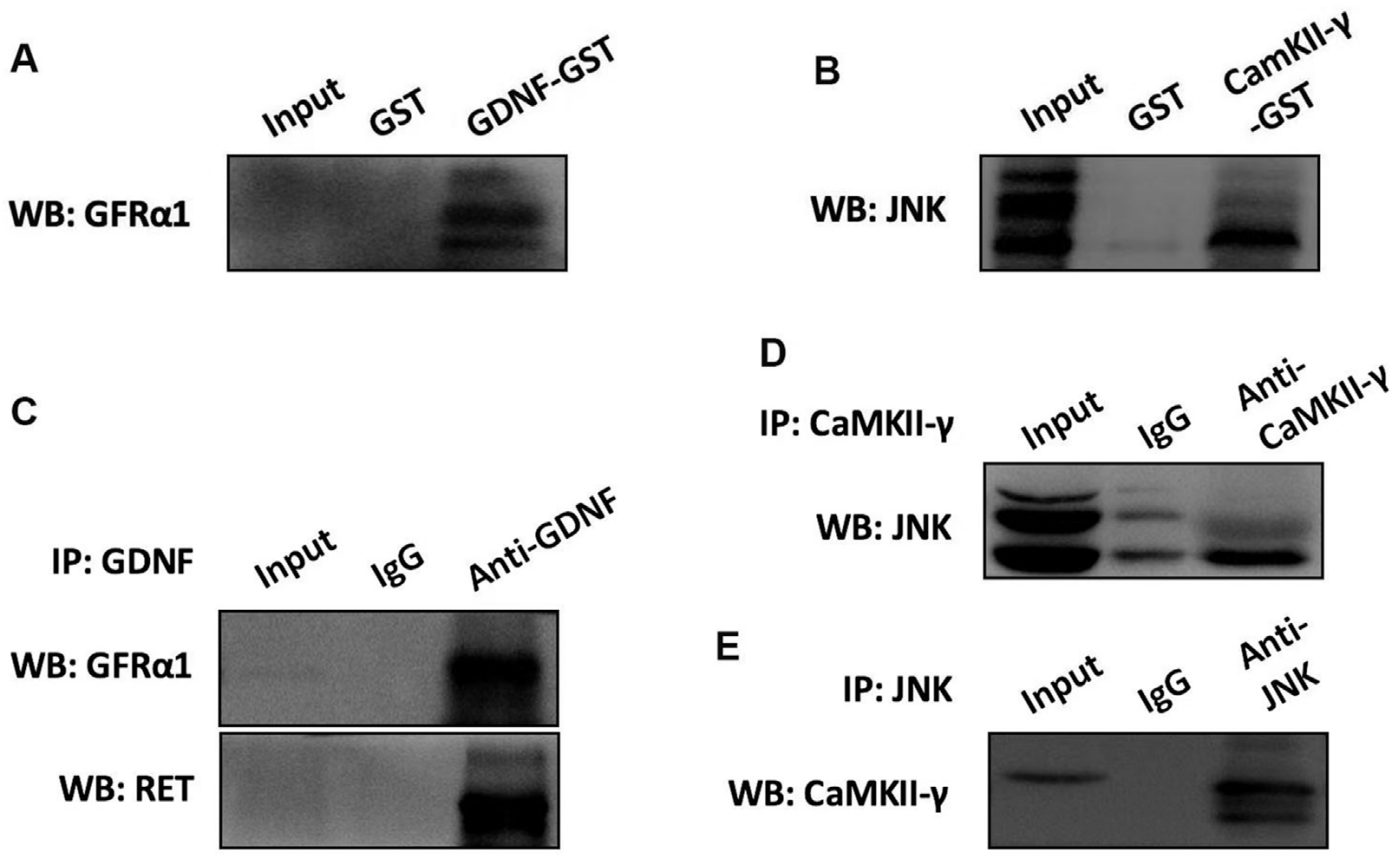
The interaction of GDNF and GFRα1/RET, CaMK II and JNK in RBL-2H3 cells. **(A)** GST pull-down assay using recombinant GST-GDNF protein confirms direct binding to the GFRα1. **(B)** GST pull-down assay demonstrated the direct binding between CaMK II and JNK. **(C)** The results of Co-IP indicated that in RBL-2H3 cells, GDNF and GFRα1/RET interact with each other. **(D,E)** The results of Co-IP Confirms CaMK II and JNK interact with each other in RBL-2H3 cells.

To investigate the interaction between GDNF and GFRα1/RET, CaMK II and JNK in RBL-2H3 cells, the GST-pull down and co-immunoprecipitation (Co-IP) were conducted. The results showed that there were interactions between GDNF and GFRα1/RET, as well as CaMK II and JNK (Figure 6).

## 4 Discussion

It is widely acknowledged that the immune function of the intestine is intricately regulated by a various of immune cells. Among these, mast cells, recognized as one of the most critical immune cells in the gastrointestinal tract, play a pivotal role in the onset and progression of intestinal inflammation. Araki Y et al. (Araki et al., 2000) discovered that rats with mast cell deficiency were unable to develop colitis induced by DSS. Also in this classic animal model for IBD, researchers found that the number of degranulated mucosal mast cells, rather than an increased number of mucosal mast cell themselves, was associated with DSS-induced intestinal inflammation (Iba et al., 2003). Thus, inhibiting mast cell degranulation appears to be a promising strategy for the treatment of IBD. In the present study, we found that GDNF can inhibit the release of β-hexosaminidase and inflammatory cytokine from mast-cell-like RBL-2H3 cells, which are triggered by exogenous stimuli. This indicated that GDNF may be a candidate therapeutic drug for treating IBD.

As a secreted protein, GDNF needs to mediate its signal transduction through a receptor complex comprised of two subunits (Paratcha and Ledda, 2008). One subunit is involved in ligand binding, the glycosyl-phosphatidylinositol (GPI)-anchored co-receptor GFRa, and the other is specialized in transmembrane signaling, the classic receptor tyrosine kinase Ret and the subsequently found neural cell adhesion molecule (NCAM) (Paratcha et al., 2003). Our previous study was the first to report that GFRα1/RET is expressed in mast-cell-like RBL-2H3 cells (Xie et al., 2020). In the current study, we have further confirmed that the inhibitory effect of GDNF on mast-cell-like RBL-2H3 cells activation was mediated through the GFRα1/RET receptor complex. The supporting evidence includes immunofluorescence co-localization demonstrated the presence of both GDNF and GFRα1 on the cell membrane; a protein interaction assay confirmed the binding of GDNF to the GFRα1/RET complex; and the effect of GDNF was effectively blocked by a specific RET inhibitor. The activated Ret protein tyrosine kinase subsequently activates intracellular proteins and initiates a series of signaling pathways, including Mitogen-activated protein kinase (MAPK), phosphatidylinositol-3-kinase (PI3K) and Phospholipase C (PLC) pathways, which regulate the release of Ca^2+^ and other biological processes (Paratcha and Ledda, 2008; Lundgren et al., 2012; Veit et al., 2004).

Intracellular Ca^2+^ is essential for mast cell degranulation. Studies have shown that inhibition of extracellular calcium influx can reduce mast cell degranulation and release of inflammatory factors, suggesting a link between Ca^2+^ and mast cell degranulation (Zhang et al., 2024; Liu et al., 2022). Subsequent research has revealed that the Ca^2+^ needed for degranulation came from the release of Ca^2+^ from intracellular stores and Ca^2+^ influx from extracellular medium through plasma membrane channels (Ma and Beaven, 2011; Chen et al., 2017). When activated by antigens and other stimulants, mast cells trigger the release of Ca^2+^ stored in the endoplasmic reticulum through PLC-mediated production of inositol1,4,5-trisphosphate, and subsequently induce extracellular Ca^2+^ influx to maintain elevated levels of cytosolic Ca^2+^ concentration. In previous studies involving other cell types, it was observed that the level of intracellular Ca^2+^ increased following the phosphorylation of the RET protein induced by GDNF (Rusciano et al., 2010; Lundgren et al., 2012; Pérez-García et al., 2004). Intriguingly, contrary to previous findings, our study demonstrated that using exogenous GDNF on already activated mast-cell-like RBL-2H3 cells resulted in a significant reduction in Ca^2+^ influx. The reason may be related to the inhibitory effect of GDNF on calcium overload. Studies have shown that GDNF could reduce the accumulation of Ca^2+^ in nerve cells during the process of secondary spinal cord injury, thereby mitigating ischemic injury to spinal cord tissue (Song et al., 2001). *In vitro* experiment have also showed that pretreatment with GDNF could effectively decrease the elevation of intracellular Ca^2+^ level induced by glutamate, thereby attenuating the excitotoxic neuronal death caused by glutamate (Nicole et al., 2001).

The activation of intestinal mast cell extends beyond merely mast cell degranulation to encompass the release of inflammatory cytokines (Gilfillan and Tkaczyk, 2006). Our research indicates that GDNF can inhibit the release of inflammatory cytokines such as IL-1β, IL-6, and TNFα. To further characterize the suppression mechanism of GDNF on mast cells activation, we investigated the expression of downstream signaling molecules involved in the release of inflammatory cytokines. There is a close association between JNK and mast cell activation. In mice model of passive cutaneous anaphylaxis, a JNK1/2-specific inhibitor blocked IgE-mediated mast cell activation and cytokine release (Wang et al., 2021). *In vitro*, activated RBL-2H3 cells induced the expression of inflammatory cytokines (TNF-α and IL-4) through the activation of JNK (Kim et al., 2019). Our results showed a significant reduction in phosphorylated JNK protein levels in mast-cell-like RBL-2H3 cells treated with GDNF, accompanied by decreased cytokine release. There was no statistical significance in the mRNA and protein expression of total JNK and its isoforms (JNK1, 2, 3) between the groups, which demonstrated that GDNF’s effect on JNK primarily involves modulating its phosphorylation/dephosphorylation status rather than altering the expression of these isoforms.

The Ca^2+^/CaMK system has been recognized as an intracellular information transmission system. Our experiments demonstrated the presence of CaMKII in the cytoplasm of mast cell-like RBL-2H3 cells and interacts with JNK, which is also localized in the cytoplasm. Besides, consistent with previous studies (Lu et al., 2020; Zhou et al., 2018), we also found that CaMKII RNAi could downregulate the expression of p-JNK and reduce mast-cell-like RBL-2H3 cells degranulation and inflammatory cytokine release. However, GDNF did not affect CaMKII mRNA and protein expression based on our findings. Thus, we hypothesize that GDNF may indirectly regulate p-JNK expression by modulating CaMKII function through the reduction of intracellular Ca^2+^ concentrations. This hypothesis is supported by the experimental evidence that, in the presence of CaMKII shRNA-induced suppression, the addition of GDNF unable to further decrease the expression of p-JNK or inhibit mast-cell-like RBL-2H3 cells degranulation, suggesting that the effects of GDNF are mediated through its influence on CaMKII function.

Our research may have some limitations. For instance, although the RBL-2H3 cell line provides a controlled system for studying FceRI-mediated mast cell degranulation, it cannot fully replicate the complex multicellular interactions and microenvironment of the *in vivo* intestinal tissue in IBD. Secondly, the precise mechanism by which GDNF reduces the intracellular Ca^2+^concentration remains to be elucidated. It is plausible that it could act by reducing Ca^2+^influx through membrane channels or by inhibiting Ca^2+^release from the endoplasmic reticulum. In addition, our research focus on the early-phase pro-inflammatory mediators (such as IL-1β, IL-6, and TNF-α), which exert pro-inflammatory effects in the intestine and act as mediators of mucosal damage. However, other inflammatory cytokines assioatated with IBD, such as IL-4, were not evaluated in this study. Thus, our findings are informative but preliminary; the proposed mechanisms require further investigation in more physiologically relevant models.

In summary, our study demonstrates that GDNF reduces the intracellular Ca^2+^ concentration in activated mast-cell-like RBL-2H3 cells by binding to GFLα1/RET receptors expressed on the cell membrane, thereby further inhibiting the Ca^2+^-mediated degranulation and the Ca^2+^/CaMKII/JNK pathway for inflammatory cytokine release. This study provides new insights into the molecular mechanism by which GDNF regulates intestinal mast cells, and provides a valuable foundation for further basic research and clinical applications of GDNF in IBD.

## Data Availability

The raw data supporting the conclusions of this article will be made available by the authors, without undue reservation.
