# Supplementary material for: Glial cell line-derived neurotrophic factor inhibits mast-cell-like RBL-2H3 cells activation via Ca2+-mediated degranulation and Ca2+/CaMKⅡ/JNK pathway
Source: Front Pharmacol. 2025 Nov 18;16:1697815. doi: 10.3389/fphar.2025.1697815 (PMC12669010; doi:10.3389/fphar.2025.1697815)
Supplement: Supplementary file 1 [file Table1.docx]

| Supplementary Table 1. The primers of relevant signaling molecules in RBL-2H3 cells | | |
| --- | --- | --- |
|  | FP | RP |
| JNK | 5’-TCCAGTTCTCGTACCCGCTA-3’ | 5’-AGCATGGCGTGACACAGTAA-3’ |
| JNK1 | 5’-TTTGGTACCGACACCAGCTC-3’ | 5’-ACCCCAGGTCCGATAAGTCA-3’ |
| JNK2 | 5’-CAGCCTTCAGATGCAGCAGT-3’ | 5’-CGCAGGCAATCCTACTGGAA-3’ |
| JNK3 | 5’-TGATGGAACTGATGGACGCC-3′ | 5’-TCCACGTTCTCCTTGTAGCC-3′ |
| GFRα1 | 5’-GGTCATCAAGCGTCTCCTGT-3’ | 5’-TTTCAGGGCTCAATGGAGGA-3’ |
| c-RET | 5′-GCTCTATGACGATGGGCTCT-3′ | 5′-TCTCGTGAGTGGTACAGGAC-3′ |
| CaMKII | 5′-ATGGCCACCACCGCCACCT-3′ | 5’-CTGCAGCGGTGCGGCAGGG-3’ |
| Actin | 5′-GTACAACCTTCTTGCAGCTCCTC-3′ | 5’-GTCCTTCTGACCCATACCCA-3’ |
