# Supplementary figures and images for: Glial cell line-derived neurotrophic factor inhibits mast-cell-like RBL-2H3 cells activation via Ca2+-mediated degranulation and Ca2+/CaMKⅡ/JNK pathway
Source: Front Pharmacol. 2025 Nov 18;16:1697815. doi: 10.3389/fphar.2025.1697815 (PMC12669010; doi:10.3389/fphar.2025.1697815)

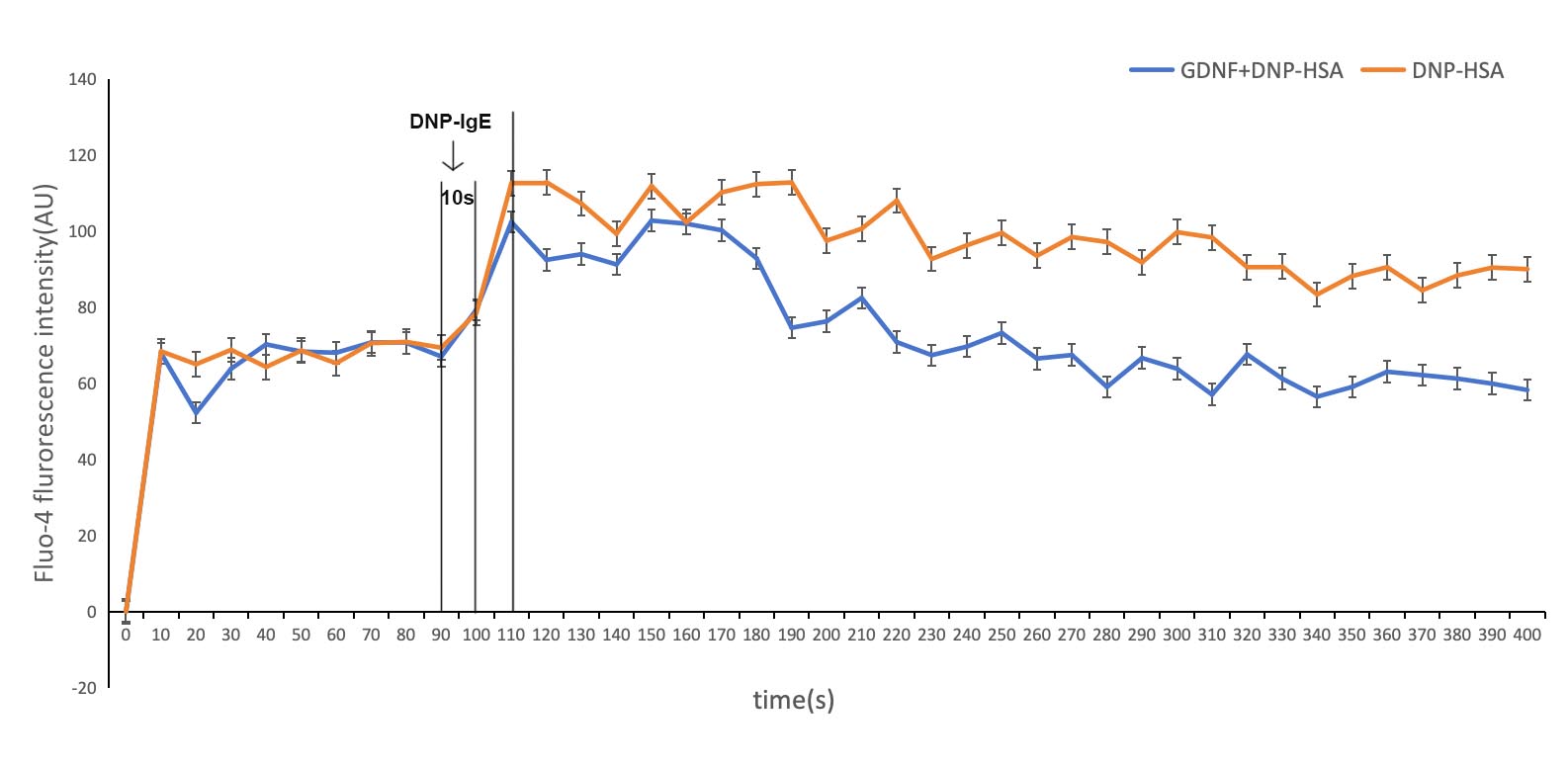

Supplement: Supplementary file 2 [file Image1.jpeg]

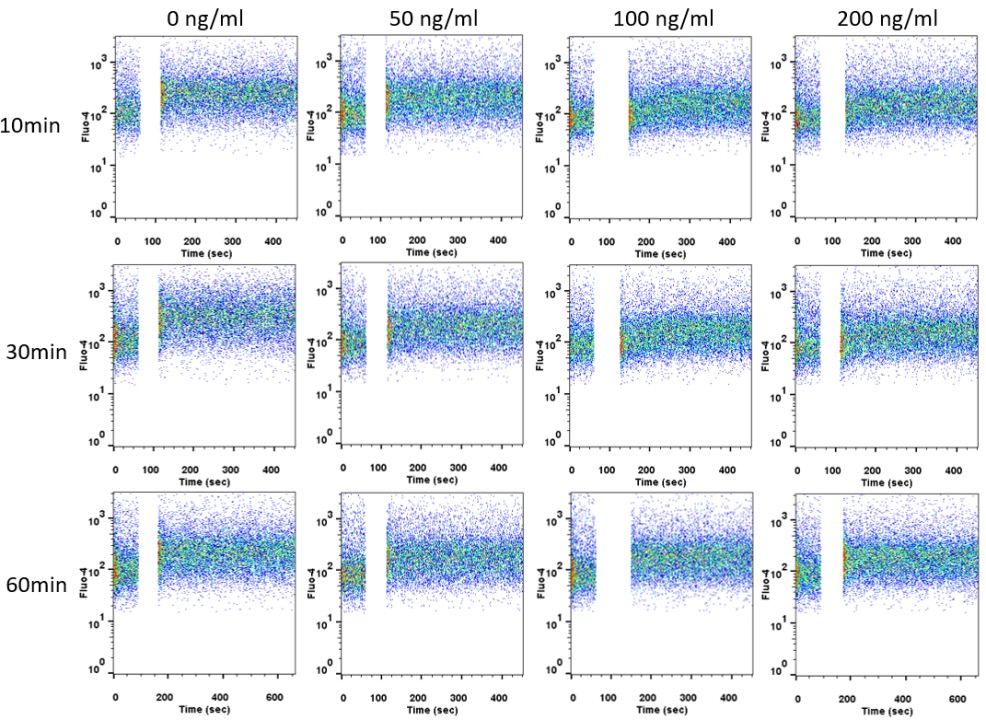

Supplement: Supplementary file 3 [file Image2.jpeg]
